# “It is not the fault of the health care team - it is the way the system works”: a mixed-methods quality improvement study of patients with advanced cancer and family members reveals challenges navigating a fragmented healthcare system and the administrative and financial burdens of care

**DOI:** 10.1186/s12913-024-11744-z

**Published:** 2024-11-11

**Authors:** Ellis C. Dillon, Meghan C. Martinez, Martina Li, Amandeep K. Mann-Grewal, Harold S. Luft, Su-Ying Liang, Natalia Colocci, Steve Lai, Manali Patel

**Affiliations:** 1 Center on Aging, UConn Health, 263 Farmington Avenue, Farmington, CT 06030-5215 USA; 2 Sutter Health Center for Health Systems Research and Palo Alto Medical Foundation Research Institute, Sutter Health, Palo Alto, CA USA; 3 (Formerly) Sutter Health Center for Health Systems Research and Palo Alto Medical Foundation Research Institute, Palo Alto, CA USA; 4 Philip R. Lee Institute for Health Policy Studies, University of California San Francisco, San Francisco, CA USA; 5 Medical Oncology, Palo Alto Medical Foundation, Palo Alto, CA USA; 6 Palliative Care and Support Services, Palo Alto Medical Foundation, Palo Alto, CA USA; 7 Medical Oncology, Stanford University, Palo Alto, CA USA

**Keywords:** Advanced cancer, Healthcare fragmentation, Patient and family member experiences, Mixed-methods, Qualitative, Financial burden, Administrative burden

## Abstract

**Background:**

Healthcare fragmentation and lack of care coordination are longstanding problems in cancer care. This study’s goal was to provide in-depth understanding of how the organization and fragmentation of healthcare impacts the experiences of patients with advanced cancer and their families, especially near the end-of-life.

**Methods:**

This mixed-methods quality improvement study took place at a large multi-specialty healthcare organization in Northern California. Electronic health record data was used to identify patients with advanced cancer and their characteristics. Data were collected 10/2019-05/2022 through periodic patient surveys and in-depth interviews with sampled family members, including open-ended questions about overall healthcare experiences. Data were analyzed using inductive thematic analysis.

**Results:**

Overall, 281/482 (58.3%) patients with advanced cancer completed surveys. Surveyed patients’ mean age was 68 (SD: 12.8) years, 53% were male, 73% White, 14.2% Asian, 1.4% Black, 3.9% Other; 8.9% Hispanic, and 19.2% were deceased within 12 months. Twenty-four family members completed in-depth interviews: 17/24 (70.8%) were spouses, 62.5% were female and 50% were interviewed after the patient’s death. Respondents were generally positive about health care team interactions, but consistently brought up the negative impacts of the *organization* of healthcare, “It is not the fault of the health care team - it is the way the system works.” Three major challenges were identified. (1) Systemic healthcare care fragmentation, including difficulties navigating care across providers and institutions, “*It seems like everything is like an isolated incident… there’s no overall*,* big picture viewpoint*.” (2) Administrative burdens, “*In the end I gave up [scheduling care] because I was tired of calling*.” (3) Financial burdens, “*This oncologist wanted to put him on a drug…but it was $4000 a month.”* Respondents described these challenges leading to worse quality of care, health, and quality of life, and loss of trust in the national healthcare system.

**Conclusions:**

These findings illustrate how care fragmentation and administrative and financial burdens lead to worse quality care and distrust of healthcare. Better coordination of patient-centered care, and a fundamental restructuring of a highly fragmented national healthcare system are required to meet the needs of patients with complex conditions like advanced cancer and their families.

**Supplementary Information:**

The online version contains supplementary material available at 10.1186/s12913-024-11744-z.

## Introduction

Research aimed at improving the delivery of health care has uncovered many barriers and facilitators of care at the level of the interaction between the patient and physician, and with respect to the organization of care within clinics, hospitals, and health care organizations [1, 2]. However, delivery of care for individuals with complex conditions like cancer typically requires interactions with many health care institutions with differing responsibilities including hospitals, ambulatory care clinics, pharmacies, insurance companies, and infusion centers. Little is known about the challenges patients and families face transitioning between and bridging these various institutions involved in different elements of their care.

Fragmentation of care, i.e., when care is spread across poorly coordinated providers and organizations, is common [3, 4]. For the general population, fragmented care is associated with negative outcomes including increased health care utilization, emergency department (ED) visits, hospitalizations, costs, and decreased patient satisfaction, and may be more pronounced for patients with chronic conditions [3–6]. People with advanced cancer are particularly at risk, given that many are older adults who receive care from numerous specialists and across multiple clinics and institutions [7]. Despite improvements in cancer prevention and treatment, delivering high-quality care, especially near the end-of-life, remains challenging [7, 8]. Another notable challenge for patients and families is the high cost of care and out-of-pocket costs leading to “financial toxicity” or financial problems due to cancer care [9]. One study found financial toxicity to be the strongest predictor of worse quality of life for patients [10]. Other recent studies have begun to explore the role of “administrative burden” in determining important patient outcomes, including delivery of care and quality of life. Administrative burden has been shown to negatively affect access to resources [11]. One general population study found 24.4% of survey respondents reported delaying or foregoing care due to administrative tasks, a problem likely magnified with advanced cancer care [12]. It is also likely that administrative burdens intersect with and overlap financial burdens patients face.

Most cross-sectional research and quantitative analyses of cancer care do not explicitly address challenges with the overall organization of healthcare. Organizational challenges bridge many institutions and emerge over time, with many issues surfacing near the end-of-life. Existing studies have often focused on the microsystem where patients and families interact with medical teams, a small part of the overall experience of the cancer journey [13], and overlooked the influence of organizational structures. Other studies have focused on the impact of fragmentation among specific groups of cancer patients, for example women, people with specific types of cancer, or within the Veterans Health Administration, often using large secondary datasets to examine associations with outcomes [14–17]. The limited existing qualitative work, often from outside the U.S., has documented substantial barriers to navigating care across providers and organizations [18–23]. Research within the U.S. finds that people with cancer in rural areas may face high fragmentation and challenges with coordinating care [24]. However, more work is needed to understand the breadth and depth of the impact of fragmentation on cancer care in the U.S., including at the end-of-life. This study’s goal was to provide in-depth understanding of how the organization and fragmentation of healthcare impacts the experiences of patients with advanced cancer and their families, especially near the end-of-life.

## Methods

This mixed-methods quality improvement study combined electronic health record (EHR) data with qualitative data from patient surveys and in-depth interviews with family members. A not-for-profit healthcare organization in Northern California, the Palo Alto Medical Foundation (PAMF), began a quality improvement initiative in its medical oncology department in 2019 to improve care for individuals with advanced cancer. PAMF is a large health care organization serving more than 1 million patients in the San Francisco Bay Area. PAMF has medical oncology clinics in four geographic locations staffed by 19 medical oncologists, and with ambulatory palliative care available at each location. Patients are referred to PAMF oncologists from physicians within the group, about 90% have a Primary Care Provider (PCP) within PAMF, as well as from external community practices. PAMF patients have a wide variety of insurance coverages. Patients with advanced cancer typically receive ambulatory care, systemic therapy, and sometimes clinical trials within PAMF. For acute care they go to several independent hospitals, and some seek second opinions and clinical trials elsewhere.

Patients with advanced cancer were identified from structured data fields indicating stage IV disease at diagnosis or distant metastases in the Epic EHR between 8/12/2019-10/23/2020, with the index date being the first documentation of advanced cancer. Exclusion criteria included hematologic or gynecologic cancers, as these patients were co-managed in other departments. Additional patient characteristics retrieved from EHR data included self-reported: age, race, Hispanic ethnicity, sex, marital status, primary spoken language, and from the organizational tumor registry, cancer stage and cancer site. Patient deaths prior to 3/1/2022 were identified using Social Security Administration death file data and data recorded in the EHR by providers.

The study team, including a sociologist (ECD), health economist (HL), two oncologists (NC, MP), one palliative care physician (SL), and research staff (ML), developed a set of open-ended patient survey questions (Appendix 1), including: (1) Please tell us more about any problems you have experienced with the oncology care team; (2) Please tell us what our teams can do to give you the support you need; (3) Please tell us more about what information you would like to receive; (4) How do you feel about your understanding of the treatment you are receiving? [if replied unsure] Please tell us more about what makes you unsure about this? (5) What do you hope the treatment will achieve? (6) How do you feel about the information you have about your prognosis and what to expect with your cancer over time? [if unsure] Please tell us more about what makes you unsure about this? (7) Please tell us more about your experience speaking with the oncology social worker. (8) Please share with us more about how cancer has affected your life. (9) Please tell us more about what could be done to improve your experience.

Surveys were distributed to all eligible patients at 1, 4, 8, and 12-months after the index date primarily by phone, with mail and email for people who could not be reached by phone, from October 2019 to November 2021. Patients received background information about the survey and if they agreed to participate, completed the survey. We calculated descriptive statistics to quantify patient characteristics for survey responders and non-responders and chi-square and t-tests to assess differences between groups. A p-value < 0.05 was considered to be statistically significant. All quantitative data analysis was conducted using SAS Enterprise Guide, version 7.1 (SAS Institute Inc., Cary, NC).

The study team developed a family member interview guide including questions about overall experience, diagnosis and treatment, decision-making, goal concordant care, palliative care, hospice, end-of-life care, and opportunities for improvement (Appendix 2). On a rolling basis as patients were contacted for their 4-month survey, they were asked to recommend and provide contact information for a family member familiar with their care experiences. If records indicated a patient had recently died, the interview team delayed contacting the family member for at least 2 months. Otherwise, family members were called, given a description of the project, and if they agreed to participate, completed a 30–60 min audio-recorded interview and received a $25 gift card. Outreach to family members to recruit individuals for interviews was concluded after reaching thematic saturation [25]. Interviews were completed March 2021 to May 2022 and family member relationship, gender, and time from patient death to interview (if applicable) was collected. A team of non-clinician researchers trained in qualitative methods (including MCM, ML) and led by a medical sociologist (ECD), handled recruitment, survey administration, in-depth interviews, and analysis. Along with research staff assisting with data collection, this team met weekly to discuss progress, concerns, and the potential influence of our own positions, histories, and experiences. No members of the data collection and analysis team had any prior relationships with recruited participants.

Transcripts of family member interviews and patient responses to open-ended survey questions were uploaded into Dedoose (version 9.0.54) and analyzed using a pragmatic realist approach to inductive thematic analysis to identify and categorize themes [26, 27]. The team collaboratively created and refined a codebook and two independent coders assigned codes, resolving any disagreements through team discussions to reach consensus. Analysis included categorizing codes into themes, exploring variations in experience, contrasting patient and family member perspectives, and selecting representative illustrative quotes included below. This work was deemed quality improvement by the Sutter Health Institutional Review Board. We adhered to recommended guidelines on reporting qualitative research [28].

## Results

Of 482 patients contacted, 281 (58.3%) completed one or more surveys (survey responders), totaling 585 unique surveys (153 at 1-month, 161 at 4-months, 152 at 8-months, 119 at 12-months). Survey responders’ mean age was 68 (standard deviation [SD]: 12.8) years, 53% were male, 73% White, 14.2% Asian, 1.4% Black, 3.9% Other, and 8.9% Hispanic (Table 1). Documented cancer stage was 61.2% Stage IV, 20.3% Stage 0-III with recurrence and metastases, 1.8% Glioblastoma (unstaged), and 16.7% were missing stage information. The most common cancer sites were gastrointestinal (27.4%), genitourinary (24.2%), thoracic (22.1%), and breast (16.4%). Compared to non-responders, survey responders were more likely to have English listed as their primary language (96.1% vs. 85.1%, *p* < 0.001) and were less likely to be deceased within 12 months following the date of first documentation of advanced cancer (19.2% vs. 46.8%, *p* < 0.001).

**Table 1:** Characteristics of patients with advanced cancer overall, and those who completed at least one survey, compared to non-responders

Characteristics	Overall *N* (%)	Survey Responders *N* (%)	Non-responders *N* (%)	*P*-value
**Total**	482	281 (58.3%)	201 (41.7%)	
**Age**^**a**^ Mean [SD]	68 [12.4]	68 [12.8]	69 [11.8]	0.53
**Age by Category** ^**a**^				0.63
< 65	164 (34.0%)	98 (35.9%)	66 (32.8%)	
65–74	154 (32.0%)	86 (30.6%)	68 (33.8%)	
75–79	67 (13.9%)	43 (15.3%)	24 (11.9%)	
80+	97 (20.1%)	54 (19.2%)	43 (21.4%)	
**Sex**				0.26
Female	216 (44.8%)	132 (47.0%)	84 (41.8%)	
Male	266 (55.2%)	149 (53.0%)	117 (58.2%)	
**Race**				0.07
Asian	76 (15.8%)	40 (14.2%)	36 (17.9%)	
Black	11 (2.3%)	4 (1.4%)	7 (3.5%)	
White	328 (68.1%)	205 (73.0%)	123 (61.2%)	
Other ^b^	23 (4.8%)	11 (3.9%)	12 (6.0%)	
Unknown/Prefer not to answer	44 (9.1%)	21 (7.5%)	23 (11.4%)	
**Hispanic Ethnicity**				0.21
Hispanic	42 (8.7%)	25 (8.9%)	17 (8.5%)	
Non-Hispanic	385 (79.9%)	230 (81.9%)	155 (77.1%)	
Unknown	55 (11.4%)	26 (9.3%)	29 (14.4%)	
**Marital Status**				0.06
Married/Partnered	328 (68.1%)	199 (70.8%)	129 (64.2%)	
Single ^c^	109 (22.6%)	63 (22.4%)	46 (22.9%)	
Unknown/Missing	45 (9.3%)	19 (6.8%)	26 (12.9%)	
**Primary Language**				< 0.001
English	441 (91.5%)	270 (96.1%)	171 (85.1%)	
Non-English	39 (8.1%)	10 (3.6%)	29 (14.4%)	
Unknown	2 (0.4%)	1 (0.4%)	1 (0.5%)	
**Cancer Stage** ^**d**^				0.15
Stage 0-III with recurrence	96 (19.9%)	57 (20.3%)	39 (19.4%)	
Stage IV	294 (61.0%)	172 (61.2%)	122 (60.7%)	
Glioblastoma ^e^	16 (3.3%)	5 (1.8%)	11 (5.5%)	
Missing stage information	76 (15.8%)	47 (16.7%)	29 (14.4%)	
**Cancer Type**				0.70
Gastrointestinal	141 (29.3%)	77 (27.4%)	64 (31.8%)	
Genitourinary	107 (22.2%)	68 (24.2%)	39 (19.4%)	
Thoracic	104 (21.6%)	62 (22.1%)	42 (20.9%)	
Breast	80 (16.6%)	46 (16.4%)	34 (16.9%)	
Other ^f^	50 (10.4%)	28 (10.0%)	22 (11.0%)	
**Deceased**				
Deceased within 12 months	148 (30.7%)	54 (19.2%)	94 (46.8%)	< 0.001

T-test (continuous variables) and Chi-square test (categorical) were used to compare group differences

*SD* Standard Deviation

^a^Age when first identified in electronic health record as meeting inclusion criteria. ^b^Other race includes Pacific Islander, American Indian, mixed race, and other race not specifically reported in electronic health record. ^c^Single includes ‘single’, ‘widowed’, ‘separated’, ‘divorced’, ‘legally separated.’ ^d^For those missing cancer stage in the EHR ClinicalPath system, we report stage at diagnosis in tumor registry data. ^e^If patients have glioblastoma plus other cancers, Glioblastoma would be reported as their primary diagnosis. ^f^Other cancer types include head and neck, melanoma and other skin cancers, neuro, sarcoma, thyroid, and other endocrine including thymus cancers

Eighty-two patients referred a family member, the team contacted 50 family members and 24 (48%) participated in an in-depth interview before thematic saturation was reached. Of twenty-four family members interviewed, 17 (70.8%) were spouses, 5 (20.8%) were children/children-in-law, 1 (4.2%) was a sister, and 1 (4.2%) a mother. Fifteen (62.5%) family members interviewed were female and 12 (50%) interviews occurred after the patient’s death (mean 175 days post-death) and included questions about end-of-life experiences (Table 2).

**Table 2:** Characteristics of interviewed family members

	*N* (%)
**Total family members interviewed**	24
**Sex**	
Female	15 (62.5%)
Male	9 (37.5%)
**Relationship to patient**	
Spouse	17 (70.8%)
Child/Child-in-law	5 (20.8%)
Sister	1 (4.2%)
Mother	1 (4.2%)
**Patient deceased at time of interview**	12 (50%)
Days from patient death to interview (mean [SD])	175 [126]

**Fig. 1:**
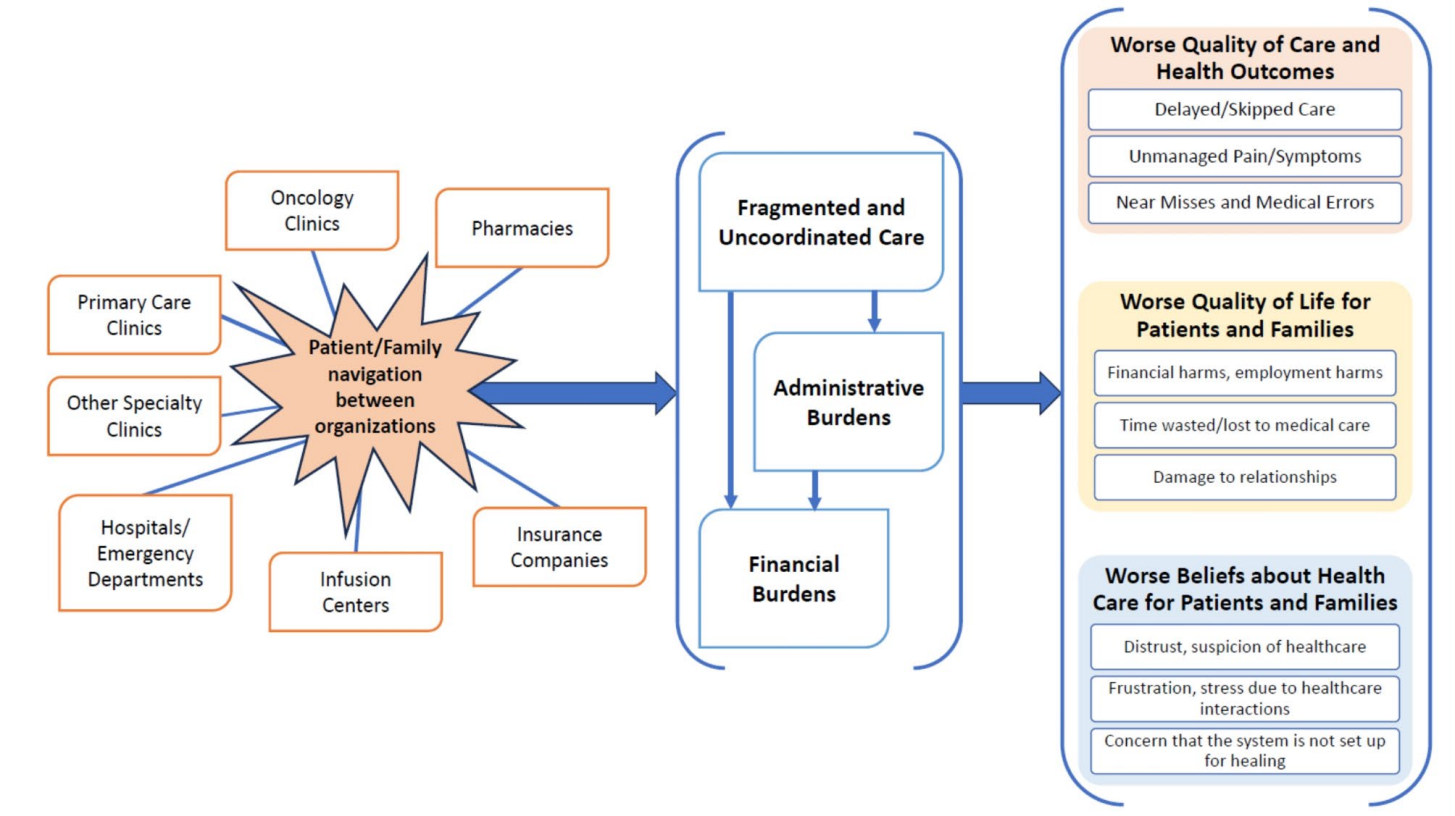
Challenges described by patients and family members in navigating care and negative impacts on quality of care, health, quality of life, and beliefs about the healthcare system

Overall, patients and family members expressed satisfaction with most aspects of their cancer care at PAMF: “I have been very happy with the care.” However, while highlighting the positive feelings about their healthcare teams, they often mentioned challenges with the organization and structure of the broader healthcare system, including difficulties navigating across providers and institutions, challenges with affording care and dealing with insurance companies, and administrative burdens shouldered by patients and family members (Fig. 1). As one patient noted in reference to physicians who could not tell her the price of a medication, “It is not the fault of the health care team - it is the way the system works” (Table 3).

### Fragmented and uncoordinated care

Patients described difficulties navigating across clinical teams, specialties (primary care, oncology, surgery, infusion, urgent care, etc.), across institutions and delivery systems (e.g., from one organization’s ambulatory practice to another system’s hospital emergency department), and across different arenas of healthcare (insurers, pharmacies, ambulatory care, hospital care, etc.). Communication between teams and institutions was a source of frustration and problems. One family member describing his wife’s healthcare journey noted how “It seems like everything is like an isolated incident,” and a lack of communication between clinical teams (Table 3). This fragmentation and discontinuity of care led to dissatisfaction with the care being provided. One family member noted frustration when there was no communication between the oncology team and the team in the hospital when his family member was hospitalized. For respondents, these concerns were more prominent when a person’s disease progressed, or they were near the end-of-life.

Patients and families were confused and frustrated with the institutional processes and practices that contributed to fragmented care. For example, one family member highlighted challenges sharing medical information between different entities. This respondent noted having to “bring down the disk [CD]” with the patient’s imaging information when seeking care between the clinic and the hospital and was surprised that the two locations could not directly “share digital information.” Another family member remarked on the redundant processes they faced at each new organization or sometimes even with each new ED visit at a hospital previously visited.

Another challenge was understanding the relative roles of primary care and oncology care and the rules about types of issues to be dealt with by each. One patient noted the challenges in scheduling timely primary care visits, the costs incurred, and frustration given their frequent medical oncology appointments, “The doctor I see the most [oncologist] knows the most about me.” While many patients and families were pleased with the healthcare they received, the high utilization of care by this group of people with advanced cancer, especially among those whose disease progressed, meant that they had ample examples of how the way the health care system is organized had stymied them.

Patients and family members also suggested potential solutions to improve care coordination. They stressed the importance of advocates, such as family members who could attend visits, ask questions, help with medications, and provide support. Many expressed a desire for help “navigating” care, with advocating for their needs, understanding opaque rules, and having access to better information on costs. The husband of a patient described how confusing it was to know who to call to address his wife’s pain and elaborated upon the need for “a health overseer” and a “big picture doctor” who could be a single point of contact. When involved, the palliative care team met these needs and was described as coordinating care, providing guidance around complex decisions, and focusing on quality of life. One family member described calling their palliative care doctor during a hospitalization saying, “We really need some advocacy here,” and noting “[Palliative care doctor] did end up calling and speaking with the hospital, and that was a great, great help.”

**Table 3:** Examples of fragmented and uncoordinated care and financial and administrative burdens reported by patients with advanced cancer and their families

**Fragmented and Uncoordinated Care**
***Uncoordinated care and confusion about who to contact***: “It seems like everything is like an isolated incident…the chemo treatments aggravate other conditions…It seems there’s no overall, big picture viewpoint, because like right now, she’s in so much pain, I don’t know who to call…there don’t seem to be any solutions…I don’t even hear anybody talking to anybody else about—or telling me who to talk to or anything.” [family member] ***Lack of collaboration between physicians***: “The various specialists that I see, never get together and discuss my case. I can imagine that seeing so many patients they never get together and discuss any individual case.” [patient] ***Lack of communication between institutions***: “My one criticism, and I can’t say it’s [oncologist’s] doing, but it’s the communication. At one point, he had a hospitalization. It was during COVID and he was really sick and he didn’t want to be there in the hospital. And there didn’t seem to be any communication between the oncology office and [hospital] – so, that’s when I called the palliative care doctor and said, we really need some advocacy here… I’m not sure whose fault – like whose court that goes into. [Palliative care doctor] did end up calling and speaking with the hospital, and that was a great, great help.” [family member] ***Redundant processes with each hospitalization and potentially unnecessary hospital transfers***: “So, in March, actually, I think that’s a really bad experience… she started to lose her strength again, right? So, then I say, “Oh, this is happening again. So, let’s go back to the [local hospital].” So, we were in the ER. So, in ER, it’s, oh, you need to check in, whatever, then they do this whole routine again. And I tell them, we’re already patients here… We even came to this hospital multiple times. I know what’s the drill, but why do you guys keep doing this over and over again? Then, when you wait there for a long time, and eventually, the doctor in the ER say, it’s a young doctor so they don’t know how to do it. They say, “Oh, this is also the tumor in the brain.” I said, “I know. So, what to do about it?” They say, “Oh, we cannot do a surgery here. It’s too dangerous.” So, they say, “Oh, let’s send her out to other hospital.” I said, “What?” They said, “Oh, usually, we send to [academic hospital].” And then they eventually sent us to [different academic hospital]. So, they sent over there and then she lived there for another four days, five days, and we do all the same checks again because that’s a new hospital, right? They go through all the things again. And they find out, “Oh, it’s a tumor there. Yeah, this is cancer.” Of course, we know. And they said, “Oh, no, you already do too much for the surgery. We cannot do it here.” I said, “What the heck is that?”… I think after two, three days, they finally got in touch with our oncologist in Palo Alto and also at [local hospital]. And they both talked and they confirmed we cannot do much. Then we send back home.” [family member] ***Lack of medical records interoperability/sharing between institutions***: “If they could have a conversation between them, it would be much easier…they should have digital information…share digital information…we always have to take, make a point of getting the information ourselves, to bring it down to [the healthcare system].” [family member] ***Problems with the role of primary care in cancer care***: “I don’t know what’s going on between my oncologist and family physician. I haven’t met my family physician in several years because I’ve been dealing with my cancer. This family physician thing isn’t working. If I’m in contact with my oncologist on a regular basis, I don’t understand why I need to see my family physician. I have to pay to see my family physician and it’s difficult scheduling an appointment. This division is rubbish. The doctor I see the most knows the most about me. It would be helpful to have someone help me navigate the system. There’s no personal knowledge or acquaintance so how can my family physician help me? My expectations are low because this isn’t the way to help people heal.” [patient]
**Financial and Administrative Burdens**
***Confusion and lack of transparency about costs of care***: “The way the medical bill works was a total shock and surprise to me… I really needed a lot of help understanding my medical bills and how the system charges money and understanding that the numbers mean nothing. No none knows how much things really cost. A lot of things didn’t make sense. This was the worst part of cancer and I’ve been dealing with cancer for 8 years.” [patient] ***Confusing insurance rules/networks lead to incurring expenses***: “[Hospital was in-network] But the emergency room itself wasn’t in our insurance, or you know, like in our network. And that was a little frustrating and no one ever told us what emergency room to go to.” [family member] ***Administrative burden of time spent by patients and families***: “I would say the difficulty is I have to find time, because I’m working. So, that’s most challenging part. But you know, everybody – you still have to work, you have family. You need to find time and to schedule, juggle everything around.” [family member] “I was trying to keep medications straight and trying to keep appointments straight. She probably had, especially toward the end, three to four appointments a week, and I was trying to make sure she got to the right ones at the right time and is she fasting, or is she not fasting? So, I think the most complicated thing that we did was just trying to keep up with the schedule.” [family member] ***Challenges with rules around refills of pain medications***: “Dr. [oncologist] has prescribed Oxycodone to help manage my pain, but since this med is under very tight control, it’s been very stressful to manage the refill and get med in time. The pharmacy is literally counting the days and number of pills at each refill. I understand Dr. [oncologist] and his team is doing everything they can to help. But it would be very helpful if more flexibility can be given for late-stage cancer patients like myself. For example, relaxing the rules a little bit so cancer patients who really depend on this med can get these meds when needed.” [patient] ***Confusing deadlines and processes***: “The two-week warning, which is a mandate for him [doctor] for the state law [End of Life Option Act]. That he couldn’t, she has to ask and then wait two weeks and then ask again before they can help with the assisted living. Not assisted living, assisted dying… You need to do a better job to let people know that if they want that get me out of Dallas, help in dying, that they need to tell them well before, way before. Because by the time [daughter] asked for it, they said, “Okay, so it’s two weeks. You’ll have to wait two weeks.” And she was barely able to get that out because she was kind of doped up. And there’s no way she would’ve been able to say, “I need this again,” in two weeks. There’s absolutely no way.” [family member]

### Financial and administrative burdens

Financial and administrative burdens associated with seeking, scheduling, and receiving care were described by both patients and families and were sometimes described as a consequence of the fragmentation of care. Financial burdens, such as high out of pocket costs, were common despite all respondents having insurance. Financial burden manifested in several ways. First, people could simply not afford certain treatments and services. The high cost of care led to difficult choices about whether to attempt to pay for certain medications or services. Treatment costs added anxiety to patients already burdened by various aspects of their cancer care, especially when charges were “hitting all at once” and they were unable “to pay the whole thing.” Having to pay out-of-pocket for prescriptions or having rejected claims were also challenges leading to anger, frustration, and “shock and surprise.” Second, it was a point of frustration and distress that the out-of-pocket cost of a medication or treatment to the patient was often unknowable in advance. One patient noted that physicians are unaware of costs, contributing to patient financial burdens, “They prescribe something that is very expensive, and they have no idea the cost.” Another patient noted, “No none knows how much things really cost,” and that medical bills were “the worst part of cancer.” Third, understanding rules and network inclusions for insurance coverage was another element of financial burden. Not understanding these rules sometimes led to incurred expenses which could have been avoided. For example, a family member recounted how an emergency department visit ended up being considered out-of-network, even though the hospital was in-network. These confusing insurance rules added a layer of complexity.

Administrative burdens included time spent scheduling and receiving medical care, time spent obtaining prescriptions and other medications, and time spent in calls to insurers to seek approvals or coverage for elements of care. Administrative burden was sometimes linked to financial burdens, as in the example of incurring costs for out-of-network emergency rooms which were confusing for patients and families to identify. Administrative burden was exemplified by patients and families spending what they perceived to be inordinate amounts of time and energy trying to get necessary and recommended care. It could be exacerbated in certain situations, as in the case of one patient who changed insurance providers during advanced cancer treatment. With this change, this person’s family member noted a significant increase in the time spent on the phone with the insurer trying to get information and approvals, and as a result, “my frustration level was off the charts.” Patients and family members also described frequent insurance denials of care, which led to significant time spent on the phone with insurance companies, and stress, despite the fact that some denials were eventually reversed. Another example of administrative burden included the time and effort needed to manage the hurdles to obtaining timely refills for pain medications, especially on the weekend. Complicated, and often invisible, rules and processes in many domains of healthcare contributed to patient and family frustration with the overall healthcare system.

### How fragmented care, and financial and administrative burdens contribute to worse quality of care, quality of life, and beliefs about healthcare

The challenges described above, related to fragmented care and financial and administrative burden, were explicitly linked by respondents to a wide variety of negative outcomes. Ultimately, these negative outcomes could be conceptually grouped into three main themes: worse quality of care, worse quality of life, and worse beliefs about health care and the healthcare system (Table 4).

Worsened quality of patient care noted by patients and families included medical errors or near misses, unmanaged pain or symptoms, and care that was delayed or skipped due to the hurdles involved receiving it. The husband of one patient recalled how fragmented care led to a near miss experience where the clinical team at a hospital did not realize the patient had implanted devices incompatible with an MRI until he mentioned it. Other patients simply opted to delay or skip care after challenges with the administrative burden of care: “I was supposed to have a PET scan and it got delayed by 4 weeks because of shuffling paperwork. In the end, I gave up because I was tired of calling.”

General quality of life was negatively impacted for some patients in ways that went beyond the immediate concerns of healthcare treatment and prognosis and often included personal relationships and financial concerns. One patient powerfully expressed this sentiment saying, “It affected every part of my life. My relationships, employment, financially — and it was awful,” and went on to note being “financially ruined.” Regarding financial burdens, one patient observed, “The care they recommended for me was incredibly expensive. It makes sense that people choose to die from cancer because they can’t afford it.” Patients and family members recounted substantial time, energy, and stress due to the burden of navigating insurance and healthcare mazes. One family member expressed concerns about her own blood pressure due to the frustration from hours of phone calls with insurance companies.

Beyond the immediate effects of these experiences, some people noted worsening beliefs about healthcare. Several patients noted a longer lasting and broader distrust and suspicion of the overall healthcare system, as in the case of one patient who called billing “evil” and “the worst part of my illness.” Another patient’s mother described how the complicated rules related to the California’s End of Life Options Act (the state’s medical aid in dying law) led her daughter to ask for it too late, given the mandatory two-week waiting period. These obstacles ultimately led to feelings that the system may not be set up for healing or supporting patients. For at least one family member, their experience built upon distrust already established with the health care system: “I’m just going to say it. My dad was a Black man in America, and a lot of Black people have a distrust of the healthcare system. And I understand why, based on like the Tuskegee experiment and so many other factors.”

The patients and family members who contributed their thoughts to this study grappled with multi-faceted concerns about the impact of the healthcare system on their health, quality of life, family finances, emotions, and trust in the healthcare system in the United States.

**Table 4:** Examples of the negative outcomes of fragmented care, and administrative and financial burdens, as reported by patients with advanced cancer and their families

**Worse Quality of Care and Health Outcomes**	
*** Delayed/Skipped Care***	“This oncologist wanted to put him on a drug…but it was $4000 a month… And [we] said, no. Not even if we had a ton of insurance could we afford that…” [family member]
*** Unmanaged *** ***pain/symptoms***	“Probably the pain management towards the end was the most stressful for me because the responsibility came on very quickly and you’re dealing with these powerful narcotics…Because she had a bowel obstruction, the only pain medication that worked was through the IV or taken orally under her tongue. It’s frustrating…we had to go through the oral, but not all of her pain meds are readily available in oral form. They have to be on special order, a special pharmacy, and lots of production, paperwork to get.” [family member]
*** Near misses and *** ***medical errors***	“I was able to take her to the hospital in San Francisco where they did the scan when we thought we were going to do radiation on her liver. And I was a little bit disappointed, actually, that they did not seem aware that she had a port, a medi port, inserted in her. And that her breast expanders, both of those, were incompatible with the MRI machines…and they were probably 20 min away from taking her to do an MRI when I had to bring it up to them, I discovered on my own that they were possibly incompatible…and when they did double check, they said, ‘Oh, you’re right…’ And that didn’t make me feel very comfortable.” [family member]
**Worse Quality of Life**	
*** Harms to relationships***, ***employment***,*** and finances***	“It affected every part of my life. My relationships, employment, financially – and it was awful. I was financially ruined and professionally I am not back to where I was. I had a positive outcome but it, cancer, was completely devastating.” [patient]
*** Time wasted/lost to medical care***	“When the pandemic started is when he pretty much switched over back onto United Healthcare [insurance]. He still kept his doctors. He didn’t have to go to different doctors. But that said, I would spend anywhere from an hour to four hours a week on the phone trying to get things to happen. Oh, yeah. Oh, wow is right. My frustration level it’s amazing – I don’t know my blood pressure – because my frustration level was off the charts. I mean, it was absolutely absurd.” [family member]
**Worse Beliefs about Health Care**	
*** Lasting distrust and *** ***suspicion about the *** ***healthcare system***	“It’s affected my life in a big way and in all aspects of life. It made me face and it gave me a serious profound encounter with the healthcare system. What I saw was so bad and awful. I got good cancer treatment and I’m pleased with that. But I’m not pleased with how the health system works. I wasn’t born here and I was surprised about the billing. The billing was the worst part of my illness. It made me face true profound evil. It really changed my life. It made me suspicious of healthcare in general.” [patient]
*** Frustration***,*** stress due to interactions with healthcare systems and entities***	“Well, one thing, it seems that there’s no like overview of the healthcare. I’m not coming from a place of blame or anything like that. Because I really am relatively unfamiliar with the healthcare industry. But, what I have noticed, since this has happened, is it seems like everything is like an isolated incident, pretty much. Now, I understand the overall thing. But some of the things that have happened is that the chemo treatments aggravate other conditions. And that’s what’s happening now. And that’s what’s been happening. That’s what spurred this surgery she had, two days ago. Some kidney deal showed up that – I don’t know all the details. But she had to have a stent put in her kidney. And she’s still suffering horribly, today, from it. But so that’s an example of what I’m saying. There’s been a couple of others. It seems there’s no overall, big picture viewpoint. Because like, right now, she’s in so much pain, I don’t know who to call. Her – I don’t know the titles – her doctors know about this. But there didn’t seem to be any solutions, or, at least, I’m not hearing any. I don’t even hear anybody talking to anybody else about – or telling me who to talk to or anything. Anyway, that’s kind of where I’m at. That’s my main I wish I had some insight into.” [family member]
*** Concern that the healthcare system is not set up for healing***	“Each doctor prescribes for their own specialty and is not consulting other doctors which is not beneficial for the patient as a whole.” [patient]

## Discussion

In a quality improvement study aimed at understanding and improving experience for patients with advanced cancer in a large multi-specialty medical group in Northern California, analysis revealed that patients and families were generally very pleased with their interactions with clinicians and care within medical oncology departments. However, medical oncology care, and treatments managed by medical oncologists, were only one piece of a very complicated array of services and processes patients and families were managing. When asked to expand on problems or areas for improvement, patients and families repeatedly noted challenges with the structures, rules, and transitions between institutions, teams, and settings. Patients and family members felt challenged by fragmented and uncoordinated care and financial and administrative burdens. Moreover, they described a variety of ways in which these challenges contributed to both short and long-term negative outcomes, including stress, frustration, worse quality of life for patients and family members, near misses or medical errors, and financial burden. For some, these negative experiences led to loss of trust in healthcare institutions. The fragmented nature of healthcare for people with advanced cancer was a dominant theme, as was the lack of attention to the “big picture” of someone’s life.

Participants described how the (dis)organization of care presented obstacles, supporting other findings that fragmentation leads to increased utilization, acute care, and lower patient satisfaction. This study expands that evidence base for cancer care, illustrating the pathways by which fragmentation may lead to delayed or skipped care, medical errors or near misses, and loss of trust in healthcare, aligning with another recent study in a primary care population [29]. Clinical implications of this work include a need for navigation/care coordination support for patients; financial navigation and more transparency/availability of treatment cost information to patients; simplifying and streamlining insurance information and communication; better communication between clinical teams and institutions; interoperability of health records; and assessing patients periodically to determine what roadblocks they are encountering. These approaches may improve short-term experience of patients and family members and reduce longer-term distrust and suspicion of healthcare.

Financial and administrative burdens were described as both a consequence of fragmentation and a cause of delayed care and unaddressed pain and symptoms. Among our respondents, some patients elected to not use expensive medications and expressed stress related to paying for care, even when insured. Other research finds that financial toxicity related to cancer care is associated with lower quality of life and increased depression and anxiety, and it is especially problematic for patients with advanced cancer [9, 30–34]. Some health systems have introduced patient navigators, lay health workers, or financial navigators to support patients and family members [35–37], in efforts to help patients navigate through what can feel like a labyrinthian healthcare system. In response to concerns identified in this quality improvement project, the medical oncology clinics at PAMF are hiring financial navigators to assist patients and families in this domain. They also expanded outreach by social workers to help patients with advanced cancer develop earlier connections to palliative care teams.

Administrative burdens are less explored generally but are likely magnified with advanced cancer, the 2nd most common cause of death in the U.S [38]. Two thirds of adults in this study were 65 years or older, and many were likely juggling care for multiple chronic conditions or dealing with functional impairments unrelated to their cancer [39], increasing the likelihood of fragmented care and the burden of administrative and financial challenges. Understanding the “time toxicity” of cancer care, defined as the total time spent coordinating, receiving, and following-up on medical care, may be a critical next step for future research and quality improvement, as will understanding its impact on care decisions and outcomes [40]. This study points to the need to better measure all the downstream impacts of healthcare on both patient and family quality of life and financial well-being. Because administrative burdens are not distributed equally, but depend on resources like time, literacy, family support, financial means, etc. [11], they may also be a driver of disparities in cancer care access and outcomes. Type of insurance, for example traditional fee-for-service plans versus health maintenance organization (HMO) plans, or transitions in insurers [41, 42], may influence care fragmentation, and financial and administrative burden. Given the potential for these experiences to damage trust in healthcare, interventions are needed to enable healthcare institutions to build, engender, and retain trust.

Addressing the challenges described by these patients and family members and reducing fragmentation and bureaucracy in healthcare will require national attention and policy changes. Meanwhile, efforts to provide navigation and enhanced support through this complex system could be targeted at the individuals with the most complex needs. Value-based payment models may provide some additional incentives for care coordination, but treatment options, complexity, and costs continue to grow [43, 44], as does the prevalence of cost-sharing and underinsurance [45, 46]. Early introduction of palliative care is another important clinical intervention and was described by respondents as helping coordinate and manage the complexity of care that spanned multiple providers, institutions, and conditions. Palliative care clinicians also acted as advocates for patients and families during challenging experiences. Advanced cancer raises questions about the goals of clinical care when end-of-life is near, engages family members in complex care coordination and decisions, and exposes fragmentation and administrative and financial burdens and their consequences. Other progressive and complex illnesses may be similar, and the experiences of patients with these conditions and their families may reveal ways in which the health care system fails the growing population with complex issues who account for a disproportionate share of overall health care use [47].

### Limitations

Patients and family member participants from this health system in Northern California were likely to have higher education and income than nationwide averages, suggesting the challenges described here may be more acute in other populations. Participants reflected the clinic and local demographics which include fewer Black and more Asian individuals than nationwide averages. There was under-representation in our surveys of patients for whom English is not their primary language and of patients with shorter survival; both groups likely face added barriers to care. Patients with advanced cancer who chose not to receive any treatment would not have been identified in this cohort and therefore would be omitted from this study.

## Conclusion

This mixed-methods study found that challenges emerge at different levels of care and cross the boundaries of delivery systems, hospitals, insurers, and other healthcare entities. These findings clearly associate health care system fragmentation and financial and administration burdens experienced by patients and families with worse quality of care, health, and quality of life, and loss of trust in the national healthcare system. Strategies for overcoming these challenges include a fundamental restructuring of a highly fragmented national healthcare system and identifying patients most in need and providing supplemental services to ensure patient-centered care and quality of life.

## Electronic supplementary material

Below is the link to the electronic supplementary material.

Supplementary Material 1.

## Data Availability

The datasets generated and analyzed for this study are not publicly available due to a lack of permission from the health system to share the qualitative data or electronic health record data used in this study.

## Abbreviations

ED: Emergency Department
EHR: Electronic Health Record
PCP: Primary Care Provider
PAMF: Palo Alto Medical Foundation
HMO: Health Maintenance Organization
