# Supplementary material for: “It is not the fault of the health care team - it is the way the system works”: a mixed-methods quality improvement study of patients with advanced cancer and family members reveals challenges navigating a fragmented healthcare system and the administrative and financial burdens of care
Source: BMC Health Serv Res. 2024 Nov 11;24:1378. doi: 10.1186/s12913-024-11744-z (PMC11552108; doi:10.1186/s12913-024-11744-z)
Supplement: Supplementary file 1 — Supplementary Material 1. [file 12913_2024_11744_MOESM1_ESM.docx]

## Appendix 1. Patient survey open-ended questions

| **Topics** | **Open-ended questions** |
| --- | --- |
| **Oncology team rating** | Please tell us more about any problems you have experienced with the oncology care team. |
| **Support from oncology team** | Please tell us what our teams can do to give you the support you need. |
| **Quality of information about treatment and treatment choices** | Please tell us more about what information you would like to receive. |
| **Uncertainty about wanting to understand more about Understanding** | How do you feel about your understanding of the treatment you are receiving?  Please tell us more about what makes you unsure about this [wanting to understand treatment information]. |
| **Treatment Hope** | What do you hope the treatment will achieve? |
| **Uncertainty about wanting prognosis information** | How do you feel about the information you have about your prognosis and what to expect with your cancer over time?  Please tell us more about what makes you unsure about this [wanting prognosis information]. |
| **Social Worker** | Please tell us more about your experience speaking with the oncology social worker. What did you speak about? Did you find the conversations valuable? |
| **Effects of Cancer** | Please share with us more about how cancer has affected your life. |
| **Improve Care** | Please tell us more about what could be done to improve your experience. |

## Appendix 2. Family member interview guide

**Background/caregiver experience**

1. **Can you tell me a little about yourself?**
   1. Did you live close by to [patient]? OR How long were you married/together?
   2. What brought you to the Bay Area? Or how did you meet your spouse? Or where else have you lived?
2. We know that family members can have a big role in the cancer care experience. **How were you involved with [patient]’s medical care?**
   1. [if response is vague] Can you tell me a little bit more about that?
   2. Were there any other things that you helped with?
   3. Did the things you were doing change over time?
3. We are interested in trying to make our care better for patients, but also for family members. Can you share what has been difficult for you as you’ve gone through this experience with [patient]?

**Cancer diagnosis experience**

1. **How did this cancer experience start for [patient]?**
   1. When was s/he first diagnosed with cancer?
   2. How did she/he end up finding out about the cancer?
2. Is there anything that could be improved with the experience [patient] or you had around the time of [patient]’s diagnosis?

**Treatment experience**

1. We know that depending on the type of cancer, people can go through very different types of treatments. **Can you tell me what treatments and medical care [patient] received throughout the illness?**
   1. After [patient] was diagnosed, what happened next?
   2. Did patient receive chemotherapy, surgery, or radiation? Other treatments?
   3. Did she/he ever need to go to the hospital or emergency department?
2. Is there anything that could be improved with the experience [patient] or you had around the time [patient] started receiving care?

**Decision-making**

1. **Do you feel like you and [patient] had all the information you needed to make the best decisions about [patient]’s care?**
   1. [if yes] What type of information was especially helpful to you?
      1. Can you tell me a little bit more about that?
   2. [if no] What information would have been helpful to you?
      1. Anything else?
2. **Were any treatment decisions more difficult for [patient] or you than others?**
   1. [if yes] Can you tell me more about the difficult decisions and the outcomes?
   2. Did [patient] end up deciding to stop any treatments at the end of his/her illness?
      1. [if yes] When did that happen? How did she/he make that decision?
3. **What could be done to make those treatment conversations better?**
4. **Throughout this cancer experience, do you feel like [patient] was heard and his/her concerns were addressed?**
   1. [If yes] Could you share more about some topics and concerns that were brought up?
      1. Did you bring up any concerns? Did you feel your concerns addressed?
   2. [If no] What happened?

**Palliative care and hospice**

1. Did [patient] ever meet with anyone from our palliative care team?
2. [if yes] What was your experience like with the palliative care team?
   1. What was most helpful about meeting with palliative care?
   2. Was there anything you would have liked to be different?
3. [if No] Have you ever heard of palliative care?

**End of life care**

1. Would you be willing to share with us what happened at the end of [patient’s] illness?
   1. [ask any clarifying questions about sequence of events, types of care, etc.]
   2. Did [patient] end up spending any time in the emergency department or hospital?
      1. [if yes] What happened? What was the experience like?
   3. Did [patient] spend time in any other facilities? (for example skilled nursing facility)
   4. [if yes] What was that experience like for [patient]?
   5. Did [patient] get any in home care? (for example, visiting nurses, hospice)
2. Thinking back on this now is there anything that you would have liked to go differently in those last weeks or months?
3. Did [patient] ever receive hospice care?
4. [if yes] When? What was your experience like with hospice care?
   1. Do you think [patient] started hospice at the right time?
   2. Can you tell me more about how [patient] made the decision to start hospice care?
5. [if no] Would [patient] or your family have liked more information about hospice care?

**Goal concordant care**

Now I would like to ask about [patient’s] or your preferences for care. We have heard from other people who received cancer care at our organization that depending on what is important to each person, people make very different choices about their care as their illness progresses.

1. Overall, including the care received at other places, was there ever a time you think [patient] did not get the care or treatments that he/she would have wanted?
2. [if just a yes or no response] Can you talk more about that?
3. Did [patient] or you have any conversations about his/her preferences for care with anyone from the care team?
4. [if yes] How did the conversation go?
   1. When did these conversations happen? Would you have liked to have them earlier?
   2. How could these conversations be improved?
   3. Did those discussions regarding treatment choices and preferences for care change as the illness progressed?
   4. Did anyone ever talk to [patient] about writing down his/her goals and preferences for example in an advance health care directive?
5. [if no] Would you have liked to have a conversation about what would happen if the cancer progressed?
   1. What specific topics do you think would be important to talk about?
   2. Were there any specific resources or accommodations that you would have liked to be made?

**Medical Oncology social worker interactions**

1. Did [patient] ever talk to the oncology social worker?
2. [if yes] **Can you tell me a bit more about the help that [social worker name] was able to provide to [patient] or to you and your family? What was the experience like?**
   1. What topics did the social worker talk to [patient] or you about?
   2. What was helpful about these visits?
   3. Were there any challenges?
   4. What could be done to make those conversations better?
3. [If no] **Did anyone mention to you the option to meet with a social worker?**
   1. [If introduced but never used SW] Can you tell me why you or [patient] decided not to talk to the oncology social worker?
   2. [if not introduced] Were there topics you would have liked to speak to a social worker about either for your [mom/dad, etc.] or for yourself as a family member?
      1. [If yes] Which things might have been helpful to talk to her about?

**Improving care/challenges**

1. If you could go back to the beginning of [patient]’s care received for cancer, is there anything that you would have liked to go differently either to improve things for [patient] or for yourself?
2. [If response is vague] Could you please tell me more and give me a specific example?
3. [If response is still vague] What is the biggest area for improvement? What advice would you give to a family member or caregiver who’s new to this experience?
4. [If other hospitals were mentioned] We mainly talked about care at Palo Alto Medical Foundation, but we are really interested in our patients’ entire experience of dealing with cancer. Is there anything that you would have liked to go differently anywhere else?
5. [If response mentions improving patient care] What about anything that would improve things for you as a family member?
6. [If response mentions improving family member experience] What about anything that would improve things for [patient]?

Is there anything else you feel that is important to share with me about [patient]’s experience or your experience that we haven’t talked about?
